# Clinical characteristics and prognosis in patients with neuronal surface antibody-mediated autoimmune encephalitis: a single-center cohort study in China

**DOI:** 10.3389/fimmu.2023.1213532

**Published:** 2023-12-12

**Authors:** Teng Huang, Fei Liu, Baojie Wang, Chunjuan Wang, Maolin Hao, Shougang Guo

**Affiliations:** ^1^ Department of Neurology, Shandong Provincial Hospital, Shandong University, Jinan, China; ^2^ Department of Neurology, Shandong Second Provincial General Hospital, Jinan, China; ^3^ Department of Neurology, Shandong Provincial Hospital Affiliated to Shandong First Medical University, Jinan, China

**Keywords:** autoimmune encephalitis, clinical characteristics, immunotherapy, prognosis, neutrophil-to-lymphocyte ratio

## Abstract

**Objective:**

This retrospective observational study primarily aimed to analyse the clinical characteristics of patients with neuronal surface antibody-mediated autoimmune encephalitis (AE) in China and report their prognosis after immunotherapy.

**Methods:**

Clinical characteristics, laboratory or imaging examinations, and treatment outcomes of 103 patients diagnosed with AE between 1 September 2014 and 31 December 2020 were collected. Univariate and multivariate logistic regression analyses were performed to determine the predictors of poor prognosis.

**Results:**

Overall, 103 patients were enrolled in the study. The main clinical symptoms included seizures (74.8%), psychiatric and behavior disorders (66.0%), cognitive deficits (51.5%), disturbances of consciousness (45.6%), and movement disorders**/**involuntary movements (26.2%). The distribution of clinical syndromes also differed for different AE subtypes. The efficacy rates of first-line immunotherapy for anti-NMDAR, anti-LGI1, anti-GABA_B_R, and anti-CASPR2 encephalitis were 70.2%, 92.3%, 70%, and 83.3%, respectively, and rituximab was administered to 21 patients as second-line immunotherapy, including 14 patients with anti-NMDAR encephalitis, 4 with anti-LGI1 encephalitis, 2 with anti-GABA_B_R encephalitis, and 1 with anti-CASPR2 encephalitis. Five patients with poor effect of the second-line treatment received bortezomib. According to the results of the last follow-up, 78 patients had a good prognosis (mRS 0–2), and 21 patients had a poor prognosis (mRS 3–6). The proportion of patients with a poor prognosis was significantly higher in anti-GABA_B_R encephalitis compared to the other AE subtypes (*p*<0.001). Multivariate analysis indicated that elevated neutrophil-to-lymphocyte ratio (NLR) and tumour presence were independent risk factors for poor prognosis. The regression equation of the model was logit(P)=-3.480 + 0.318 NLR+2.434 with or without tumour (with assignment =1, without assignment =0). The prediction probability generated by the regression model equation was used as the independent variable for receiver operating curve (ROC) analysis. The results showed that the area under the curve (AUC) of the prediction probability was 0.847 (95% CI, 0.733–0.961; *p* < 0.001).

**Conclusions:**

Different AE subtypes demonstrated different clinical symptom spectra throughout the disease stage. Anti-LGI1 encephalitis and anti-CASPR2 encephalitis were more sensitive to first-line and second-line treatments. Anti-GABA_B_R encephalitis had the worst prognosis among the abovementioned subtypes. The regression equation constructed using NLR and tumour presence effectively predicted the poor prognosis.

## 1 Introduction

Autoimmune encephalitis (AE) is a central nervous system disease mediated by an autoimmune mechanism and is associated with the presence of specific autoantibodies against neuronal cell surface proteins, ion channels, or receptors (1). In 1968, Corsellis et al. proposed the concept of “limbic encephalitis”. In 2005, Vitaliani et al. were the first to report a series of cases of teratoma-associated encephalitis, an immune-mediated disorder (2). In 2007, Dalmau et al. were the first to identify anti-N-methyl-d-aspartate receptor (NMDAR) encephalitis (3). In recent years, an increasing number of autoimmune antibody subtypes have been discovered with the development of neuroimmunology and antibody detection techniques, including anti-leucine-rich glioma-inactivated 1 (LGI-1) antibodies, anti-gamma-aminobutyric-acid type B receptor (GABA_B_R) antibodies, anti-contactin-associated protein-like 2 (CASPR2) antibodies, anti-α-amino-3-hydroxy-5-methyl-4-isoxazole propionic acid receptor (AMPAR) antibodies, anti-metabotropic glutamate receptor 5 (mGluR5) antibodies, and anti-dipeptidyl peptidase-like protein-6 (DPPX) antibodies (4).

Studies have shown that the early initiation of immunotherapy can greatly improve the prognosis of patients with AE. Therefore, early diagnosis and treatment of AE are crucial. However, clinicians remain too reliant on antibody testing, which often takes several days to weeks in many institutions (5). In addition, AE is a pedigree disease with multiple subtypes, and its clinical manifestations are complex and vary. Therefore, correct diagnosis of AE in the initial stage is often difficult, and a delay in diagnosis and immunotherapy affects the recovery and prognosis of patients.

Therefore, in this retrospective study, we collected and analysed the clinical data (including clinical manifestations, auxiliary examinations, treatment, and prognosis) of AE in a sample of 103 patients with multiple subtypes, compared the differences in clinical features and prognosis in each subtype, and analysed the factors affecting the prognosis of AE. In this study, we aimed to improve the awareness of these diseases among neurologists and provide supporting evidence for the diagnosis and treatment of AE.

## 2 Materials and methods

### 2.1 Patient inclusion

In this retrospective study, 103 patients diagnosed with AE were enrolled from 1 September 2014 to 31 December 2020 in the Department of Neurology of Shandong Provincial Hospital, Jinan, China. This study was approved by the Research Ethics Committee of Shandong Provincial Hospital. In reference to the diagnostic criteria suggested by Graus et al. in 2016 (5) and Chinese expert consensus of AE (2017 edition) (6), patients were included in this study based on the following criteria: (1) acute or subacute onset (<3 months) of one or more of the following symptoms: a. symptoms of the limbic system: psychiatric symptoms, memory deficit, seizure; b. encephalitis syndrome: clinical manifestations of diffuse or multifocal brain damage; c. clinical manifestations of basal ganglia and/or diencephalon/hypothalamus involvement; d. psychiatric disorder that does not qualify as a non-organic disease by a psychologist; (2) with or without CSF (cerebrospinal fluid) pleocytosis, magnetic resonance imaging (MRI) features of encephalitis, or electroencephalogram (EEG) with epileptic or slow-wave activity; (3) CSF and blood serum antibody testing positive for anti-NMDAR antibodies based on a cell-based assay (CBA)(Euroimmun, Lübeck, Germany); CSF and/or blood serum antibody testing positive for other neuronal surface antibodies (CBA): patients with positive antibodies only in the serum need to have typical clinical symptoms and/or high antibody titers (>1:32); and (4) reasonable exclusion of alternative causes.

### 2.2 Data collection and analysis

The patients’ clinical data were collected and analysed by two experienced neurologists and included the following: age at onset, sex, initial symptoms, clinical manifestations, laboratory tests, antibody tests, imaging examinations, EEG data, treatment, and outcome. Patients were followed up every three months, including outpatient visits or telephone follow-ups. The efficacy rate of immunotherapy was defined as the proportion of patients with a decrease in the modified Rankin Scale (mRS) scores (≥1) within four weeks. A good prognosis was defined as mRS ≤2, where as poor prognosis was defined as mRS ≥3.

### 2.3 Antibody identification

We used the CBA to test for antibodies in the CSF and serum of patients. The initial dilution titres of the CSF and serum were 1:1 and 1:10, respectively.

### 2.4 Statistics

SPSS (version 26.0) was used to analyse all data. Continuous variables with normal distribution were presented as “mean ± standard deviation”. Continuous variables without normal distribution were presented as “median(interquartile range)”. Categorical variables were presented as numbers (percentages) and compared using the chi-squared test. The Mann–Whitney U test was used for continuous variables without normal distribution. Multivariate analysis was performed by binary logistic regression analysis.The prognosis was considered the state variable (poor prognosis=1, good prognosis=0), and the prediction probability generated by the regression model equation was used as the independent variable for receiver operating characteristic (ROC) analysis. *P*-value < 0.05 was considered statistically significant.

## 3 Results

### 3.1 Demographic data and antibody distribution

A total of 103 patients diagnosed with AE were enrolled in this study, including 62 males (60.2%) and 41 females (39.8%), with a median age of 47 years (range, 33–58 years). Among them, 48 (46.6%) had anti-NMDAR encephalitis, 28 (27.2%) had anti-LGI1 encephalitis, 20 (19.4%) had anti-GABA_B_R encephalitis, and 7 (6.8%) had anti-CASPR2 encephalitis. In addition to the above subtypes, we admitted patients with other neuronal surface antibody-mediated AE subtypes, including anti-DPPX encephalitis, anti-AMPAR encephalitis, anti-mGluR5 encephalitis and so on. However, the number of patients with these antibody subtypes was too small to be included in our study (<3).

The sex ratio and median age were different for each AE subtype. The female ratio with anti-LGI1 encephalitis was 3.6%, which was significantly lower than that of the other subtypes (*p*<0.001). The median age of anti-NMDAR encephalitis was 34, ranging from 19–44 years, which was relatively younger than that of the other subtypes (*p*<0.001). **Table 1** provides a detailed description of demographic and clinical data.

**Table 1:** Clinical data of different antibody types.

	All (n=103)	NMDAR (n = 48)	LGI1 (n = 28)	GABA_B_R (n = 20)	CASPR2 (n = 7)
Sex (M/F)	62:41	19:29	27:1	12:8	4:3
Age	47(33-58)	34(19-44)	55(49-62)	61(50-68)	40(28-58)
Tumor (n, %)	16(15.5)	4(8.3)	0(0)	12(60)	0(0)
Clinical manifestation (n, %)					
Seizures	77(74.8)	34(70.8)	19(67.9)	18(90)	6(85.7)
Psychiatric and behavior disorders	68(66.0)	31(64.6)	19(67.9)	14(70)	4(57.1)
Cognitive deficits	53(51.5)	12(25.0)	17(60.7)	17(85.0)	7(100)
Disturbance of consciousness	47(45.6)	20(41.7)	7(25.0)	14(70)	6(85.7)
Movement disorders	27(26.2)	11(22.3)	9(32.1)	2(10)	5(71.4)
Autonomic dysfunction	10(9.7)	7(14.6)	1(3.6)	0	2(28.6)
Speech disorder	8(7.8)	7(14.6)	1(3.6)	0	0
Ventilator use (n, %)	13(12.6)	8(16.7)	1(3.6)	3(15)	1(14.3)
ICU (n, %)	12(11.7)	6(12.5)	1(3.6)	4(20)	1(14.3)
mRS score ≥2 at the peak of disease (n, %)	103(100)	48(100)	28(100)	20(100)	7(100)
CSF findings (n, %)					
Increased intracranial pressure	21/99(21.2)	16/48(33.3)	2/27(7.4)	2/18(11.1)	1/6(16.7)
Increased white blood cell count	52/99(52.5)	34/48(70.8)	5/27(18.5)	12/18(66.7)	1/6(16.7)
Increased protein in CSF	21/99(21.2)	12/48(25.0)	4/27(14.8)	4/18(22.2)	1/6(16.7)
Serum autoantibody (n, %)					
TgAb (+)	13/76(17.1)	4/32(12.5)	4/20(20)	2/17(11.8)	3/7(42.9)
TPOAb (+)	13/76(17.1)	5/32(15.6)	3/20(15)	3/17(17.6)	2/7(28.6)
ANA (+)	28/80(35.0)	6/35(17.1)	8/22(36.4)	12/17(70.6)	2/6(33.3)
Abnormal EEG results (n, %)	66(64.1)	30(62.5)	19(67.9)	12(60)	5(71.4)
Abnormal MRI results (n, %)					
Normal	56(54.4)	23(47.9)	19(67.9)	10(50)	4(57.1)
Temporal lobe and hippocampus	31(30.1)	12(25.0)	9(32.1)	8(40)	2(28.6)
Frontal lobe	8(7.7)	7(14.6)	0	0	1(14.3)
Parietal lobe	2(1.9)	2(4.2)	0	0	0
Basal ganglion and thalamus	5(4.9)	4(8.3)	1(3.6)	0	1(14.3)
Others	7(6.8)	4(8.3)	0	2(10)	1(14.3)
First-line immunotherapy (n, %)					
Corticosteroids	29(28.2)	8(16.7)	14(50)	5(25)	2(28.6)
IVIG	4(3.9)	3(6.2)	0	0	1(14.3)
Corticosteroids + IVIG	69(67.0)	37(77.1)	14(50)	15(75)	3(42.9)
Second-line immunotherapy (n, %)					
RTX	21(20.4)	14(29.2)	4(14.3)	2(10.0)	1(14.3)
Bortezomib	5(4.9)	5(10.4)	0	0	0
MMF	43(41.7)	28(58.3)	11(39.3)	4(20.0)	0
Relapse (n, %)	21/99(21.2)	7/48(14.6)	1/25(4)	12/19(63.2)	1/7(14.3)
Prognosis (n, %)					
Good(mRS **≤** 2)	78(78.8)	42(87.5)	23(92.0)	8(42.1)	5(71.4)
Poor(3≤mRS **≤** 6)	21(21.2)	6(12.5)	2(8.0)	11(55.0)	2(28.6)

M, males; F, females; ICU, intensive care unit; mRS, modified Rankin scale; CSF, cerebrospinal fluid; TgAb, anti-thyroglobulin antibodies; TPOAb, anti-thyroid peroxidase anti-bodies; ANA, antinuclear antibodies; EEG, electroencephalogram; MRI, magnetic resonance imaging; IVIG, intravenous immune globulin; RTX, rituximab; MMF, mycophenolate mofetil; NMDAR, N-methyl-D-aspartate receptor; LGI1, leucine-rich glioma-inactivated 1; GABA_B_R, gamma-aminobutyric-acid type B receptor; CASPR2, contactin-associated protein-like 2.

### 3.2 Clinical characteristics

**Figure 1:**
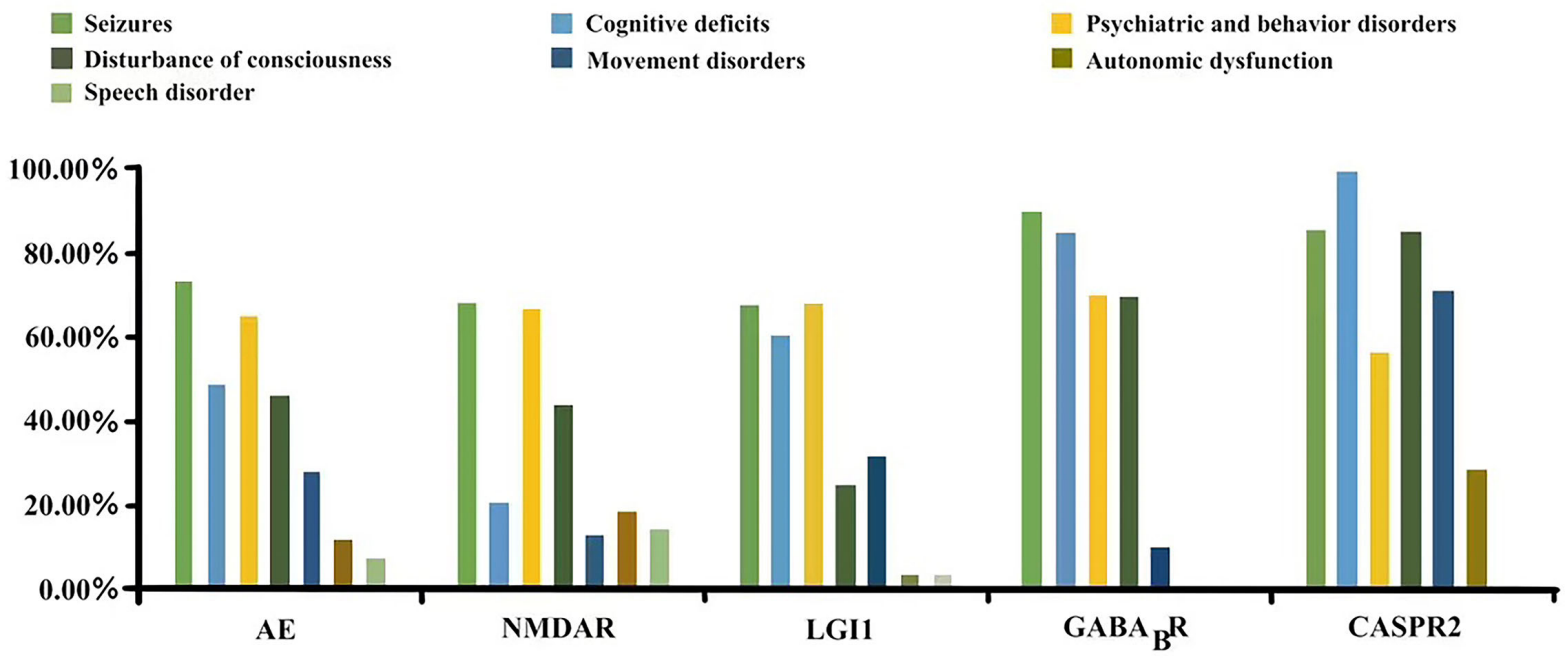
Symptom distribution of different antibody types.

Seizures, psychiatric and behavioural disorders, cognitive deficits, disturbances in consciousness, movement disorders/involuntary movement, autonomic dysfunction, and speech disorders are common clinical manifestations of AE. The distribution of clinical syndromes was different for the different AE subtypes. Anti-LGI1 encephalitis and anti-CASPR2 encephalitis had a higher percentage of patients with movement disorders compared to other subtypes. In addition, the proportion of patients with cognitive deficits was much lower in anti-NMDAR encephalitis(25.0%) than in other subtypes, which all had proportions > 60%. The proportion of consciousness disorders was much lower in anti-LGI1 encephalitis (25.0%) than in other subtypes, which all had proportions > 40%. In addition, faciobrachial dystonic seizures (FBDS) only appeared in anti-LGI1 encephalitis. The different distributions of clinical syndromes for different subtypes are shown in **Figure 1**.

All patients underwent tumour screening. Among the 103 patients, 16 patients had tumours. Four patients with anti-NMDAR encephalitis were diagnosed with ovarian teratomas, and twelve patients with anti-GABA_B_R encephalitis were diagnosed with small-cell lung cancer (SCLC). Notably, tumours were not detected at disease onset in two patients with anti-GABA_B_R encephalitis with SCLC but were found 6 months after discharge. No patient with anti-LGI1 or anti-CASPR2 encephalitis had tumour.

Twelve patients received intensive care, and thirteen patients required mechanical ventilation. Among the subtypes, the intensive care unit (ICU) admission rate of anti-GABA_B_R encephalitis was the highest (20%) and that of anti-LGI1 encephalitis was the lowest (3.6%).

### 3.3 Auxiliary examinations

**Figure 2:**
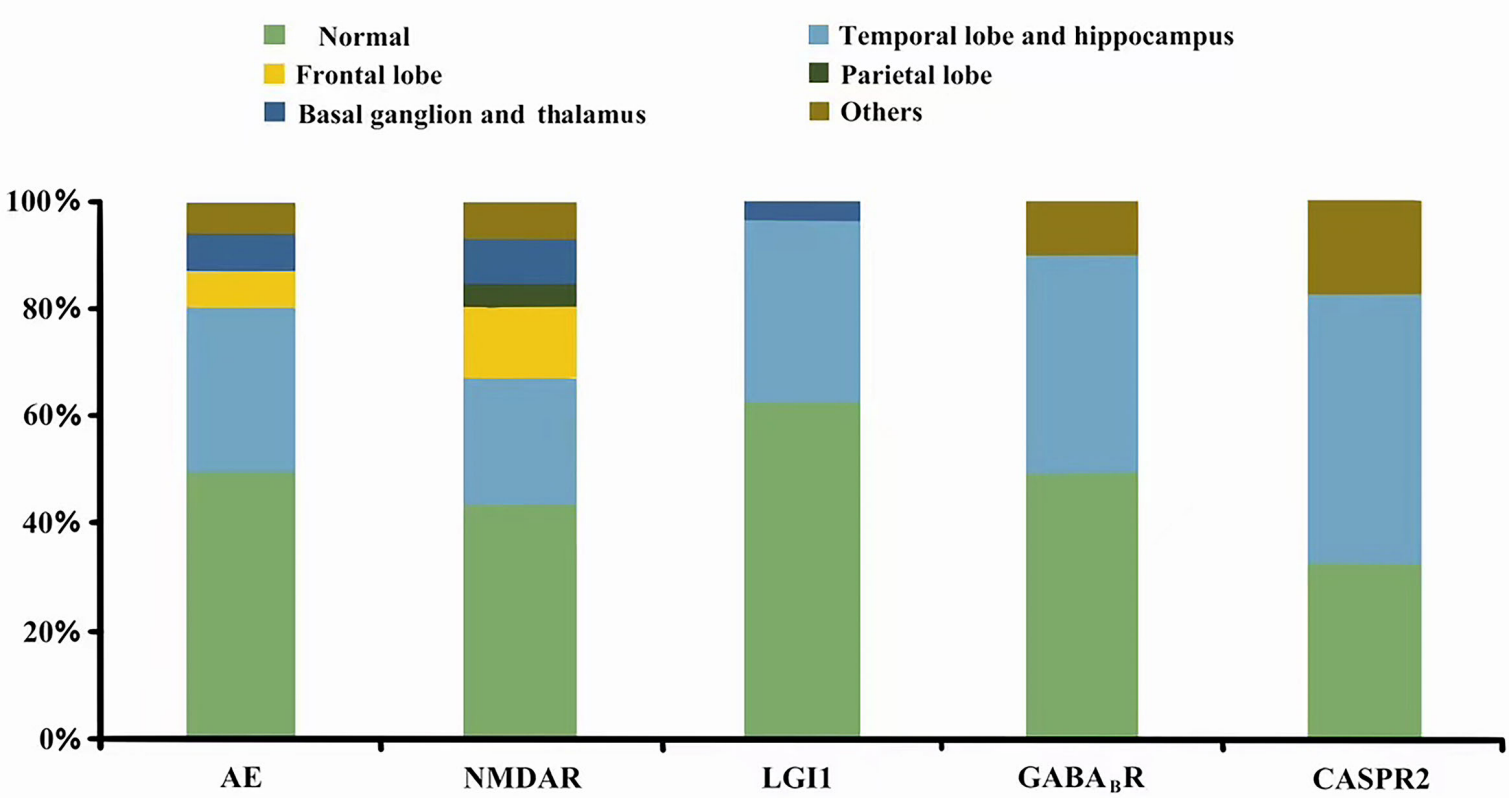
MRI features of different antibody type.

In our study, all the patients underwent brain MRI and EEG. MRI abnormalities were observed in 47 patients (45.6%), among whom the temporal lobe and hippocampus were the most commonly affected regions among all the subtypes. Other affected regions included the basal ganglion, thalamus, insula, frontal lobe, and parietal lobe. The distributions of MRI abnormalities for the different subtypes are shown in **Figure 2**. EEG abnormalities were observed in 66 (64.1%) patients, including 30 patients with anti-NMDAR encephalitis (62.5%), 19 patients with anti-LGI1 encephalitis (67.9%), 12 patients with anti-GABA_B_R encephalitis (60%), and 5 patients with anti-CASPR2 encephalitis (71.4%).The most common EEG manifestations were focal and diffuse slow waves, and epileptic discharges were observed during the seizures. The delta brush was not observed in the present study.

Ninety-nine of 103 patients had CSF findings. The other four patients refused lumbar punctures, including 2 patients with anti-GABA_B_R encephalitis, 1 patient with anti-LGI1 encephalitis, and 1 patient with anti-CASPR2 encephalitis. Including the 4 abovementioned patients who refused lumbar punctures, 14 patients were positive for antibodies only in the serum, including 5 patients with anti-LGI1 encephalitis, 5 with anti-GABA_B_R encephalitis, and 4 with anti-CASPR2 encephalitis. Of the 14 patients with positive antibodies only in the serum, 12 had high antibody titres (>1:32), whereas the antibody titres of the other 2 patients were 1:10, including one with anti-LGI1 encephalitis and one with anti-CASPR2 encephalitis. Although these two patients had relatively low antibody titers, they had very typical clinical manifestations. Overall, 52 patients (52.5%) showed pleocytosis. Increased intracranial pressure and CSF protein were observed in 21 (21.2%) patients, most of whom had slightly or moderately elevated levels.

### 3.4 Treatment and outcome

**Figure 3:**
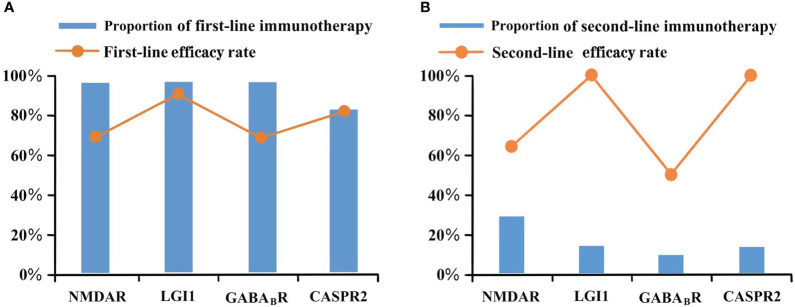
Immunotherapy of different antibody type. **(A)** Proportion of first-line immunotherapy and efficacy rate; **(B)** Proportion of second-line immunotherapy and efficacy rate.

In our study, 102 patients received first-line immunotherapy, including 29 patients who received only corticosteroids, 4 who received only intravenous immunoglobulin (IVIG, 0.4 g/kg/d; 3-5 days), and 69 who received a combination of corticosteroids and IVIG. One male patient with anti-CASPR2 encephalitis refused the first-line therapy. The patient only received an antiepileptic medication and had an mRS score of 4 at the last follow-up. The first-line efficacy rates of anti-NMDAR encephalitis and anti-GABA_B_R encephalitis were relatively low at 70.2% and 70%, respectively, and the first-line efficacy rates of anti-LGI1 encephalitis and anti-CASPR2 encephalitis were 92.3% and 83.3%, respectively. Rituximab (RTX) was administered to 21 patients as second-line immunotherapy, including 14 patients with anti-NMDAR encephalitis (14/48, 29.2%), 4 with anti-LGI1 encephalitis (4/28, 14.3%), 2 with anti-GABA_B_R encephalitis (2/20, 10%), and 1 with anti-CASPR2 encephalitis (1/7, 14.3%). Similar to the response to first-line treatment, the efficacy rates of RTX in patients with anti-NMDAR and anti-GABA_B_R encephalitis were relatively low at 64.3% and 50%, respectively, whereas the efficacy rates in the other two subtypes were 100%. **Figure 3** shows the proportion and efficacy rates of the first- and second-line immunotherapies.

Five patients with poor effect of second-line treatment received bortezomib, all of whom had anti-NMDAR encephalitis. They all had a good prognosis (mRS 0-2) at the last follow-up. Mycophenolate mofetil (MMF) was used for long-term immunotherapy in 43 patients (41.7%).

Four patients with anti-NMDAR encephalitis had ovarian teratomas, all of whom underwent tumour resection. The last follow-up showed that none of them had relapsed and all of them had good prognosis (mRS 0-2). Twelve patients with anti-GABA_B_R encephalitis were diagnosed with SCLC. Nine patients underwent anti-tumour treatment, including radiation and chemotherapy. At the last follow-up, six of the nine patients had died, one was lost to follow-up, and two had a good prognosis. All three patients without anti-tumour treatment died.

Four of the 103 registered patients were lost to follow-up, and the remaining 99 patients were followed up as outpatients or through telephone calls. The median follow-up time was 39 months (28–52 months). According to the results of the last follow-up, 78(78.8%) patients had a good prognosis (mRS 0–2), and 21(21.2%) patients had a poor prognosis (mRS 3–6). The proportion of patients with a poor prognosis was significantly higher in anti-GABA_B_R encephalitis compared to the other subtypes (*p*<0.001).

**Figure 4:**
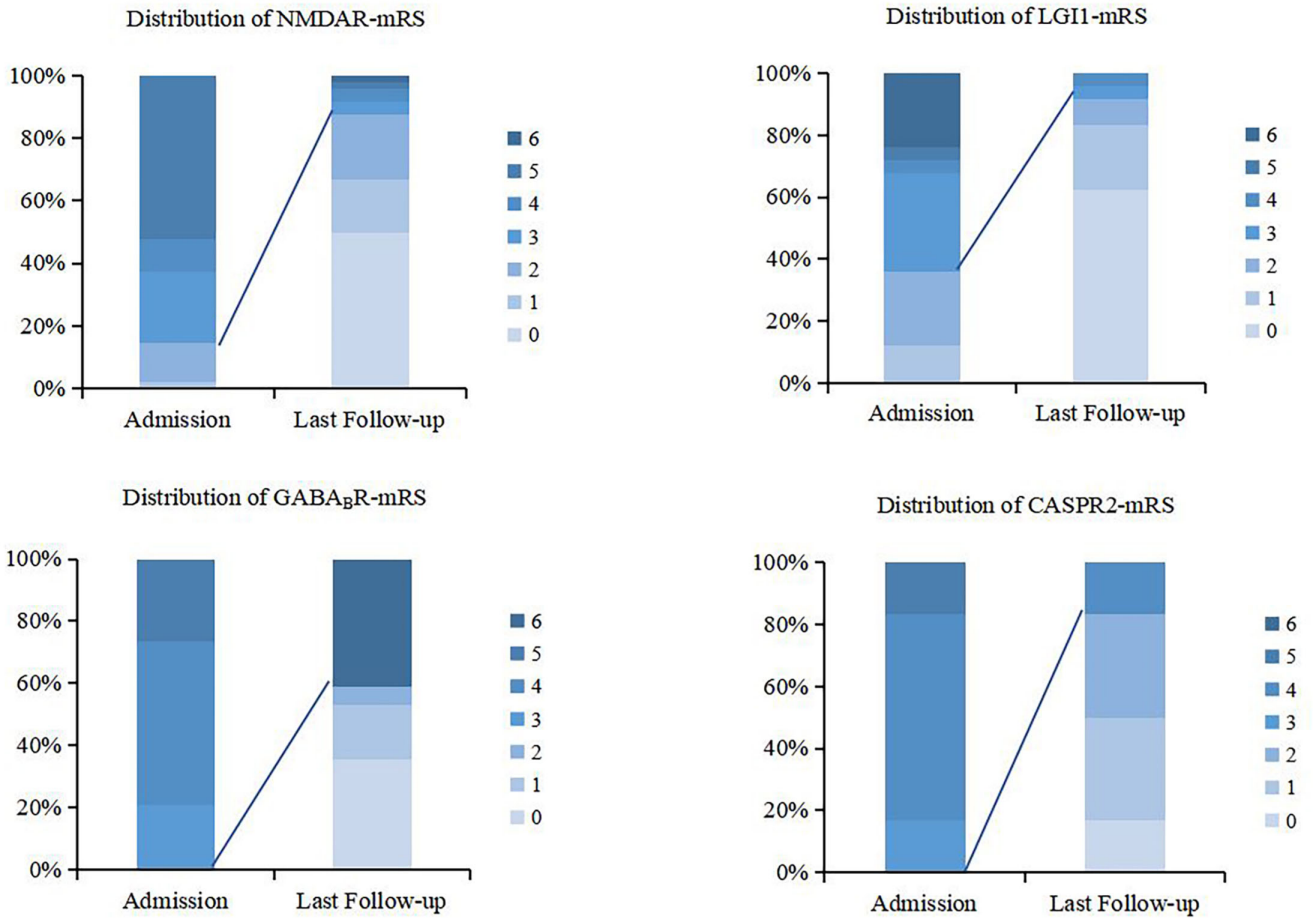
Distribution of mRS scores upon admission and the last follow-up.

Among the 21 patients with poor prognosis, 4 had mRS scores of 3, 4 had mRS scores of 4, 1 had mRS score of 5, and 12 died. The distribution of admission and last follow-up scores for the different antibody types is shown in **Figure 4**.

### 3.5 Predictors of prognosis

Among the 99 patients with AE that were followed up, excluding four patients without CSF results, we performed univariate analysis on the remaining 95 patients with complete clinical data to identify predictors of poor prognosis. Univariate analysis indicated that tumour presence (*p<*0.001), anti-GABA_B_R encephalitis (*p<*0.001), disturbance of consciousness (*p=*0.045), pulmonary infection complications (*p=*0.039), mechanical ventilation (*p=*0.030), elevated neutrophil-to-lymphocyte ratio (NLR) (*p<*0.001), and low albumin levels (*p=*0.003) were statistically significant (**Table 2**). In addition, subgroup analysis showed that elevated NLR, low albumin level, and ICU admission were risk factors for poor prognosis of anti-NMDAR encephalitis (**Table 3**). All factors with a *p*-value<0.05 in **Table 2** were included in a multivariate logistic regression model, and the entry method “Backward LR” was selected. The results showed that elevated NLR and tumour presence were independent risk factors for poor prognosis, suggesting that the risk of poor prognosis increased by 1.374 times for every 1 unit increase in NLR in patients. Furthermore, the risk of poor prognosis in patients with tumours was 11.408 times the risk in patients without tumours. According to the results of the binary logistic regression analysis, the regression equation of the model was logit(P)=-3.480 + 0.318 NLR+2.434 with or without tumours (with assignment =1, without assignment =0). **Table 4** provides a detailed description of these results.

**Table 2:** Univariate analysis of good and poor prognosis.

Variables	Total (n =95)	Good prognosis (n =76)	Poor prognosis (n =19)	*P*-value
Age (years), *M*(*IQR*)	42 (28-58)	38 (27.25-55)	50(28-65)	0.095
Sex, *n*(*%*)				0.402
Male	57 (60)	44 (57.9)	13 (68.4)	
Female	38 (40)	32(42.1)	6 (31.6)	
Tumor, *n*(*%*)	13 (13.7)	5 (6.6)	8 (42.1)	**<0.001**
Antibody, *n*(*%*)				**<0.001**
NMDAR	48 (50.5)	42 (55.3)	6 (31.6)	
LGI1	24 (25.3)	22 (28.9)	2 (10.5)	
GABA_B_R	17 (17.9)	7 (9.2)	10 (52.6)	
CASPR2	6 (6.3)	5 (5.3)	1 (1.1)	
Clinical manifestation				
Seizures, *n*(*%*)	73 (76.8)	56 (73.7)	17 (89.5)	0.248
Cognitive deficits, *n*(*%*)	49 (51.6)	36 (47.4)	13 (68.4)	0.101
Psychiatric and behavior disorders, *n*(*%*)	54 (56.8)	41 (43.2)	13 (68.4)	0.255
Disturbance of consciousness, *n*(*%*)	36 (37.9)	25 (32.9)	11 (57.9)	**0.045**
Movement disorders, *n*(*%*)	24 (25.3)	18 (23.7)	6 (31.6)	0.679
Autonomic dysfunction, *n*(*%*)	9 (9.5)	8 (10.5)	1 (5.3)	0.793
Speech disorder, *n*(*%*)	8 (8.4)	7 (9.2)	1 (5.3)	0.926
Pulmonary infection complications, *n*(*%*)	7 (7.4)	3 (3.9)	4 (21.1)	**0.039**
Mechanical ventilation, *n*(*%*)	13 (13.7)	7 (9.2)	6 (31.6)	**0.030**
Admission to intensive care unit, *n*(*%*)	10 (10.5)	6 (7.9)	4 (21.1)	0.210
CSF findings				
Increased intracranial pressure, *n*(*%*)	21 (22.1)	18 (23.7)	3 (15.8)	0.665
Increased white blood cell count, *n*(*%*)	49 (51.6)	38 (50)	11 (57.9)	0.538
Increased protein in CSF, *n*(*%*)	21 (22.1)	17 (22.4)	4 (21.1)	1.000
NLR, *M*(*IQR*)	2.97(1.99-4.43)	2.68(1.93-3.70)	5 (2.90-19.79)	**<0.001**
Albumin, *M*(*IQR*)	39.8(36.2-43.4)	40.4(36.75-44.05)	36.5(25.2-41.9)	**0.003**
Abnormal EEG, *n*(*%*)	59 (62.1)	47 (61.8)	12 (63.2)	0.916
Abnormal MRI, *n*(*%*)	44 (46.3)	36 (47.4)	8 (42.1)	0.681
Time to immune therapy (d), *M*(*IQR*)	26(12-68)	23 (12-65.5)	27 (12-74)	0.625

M,median; IQR, Interquartile Range; NMDAR, N-methyl-D-aspartate receptor; LGI1, leucine-rich glioma-inactivated 1; GABABR, gamma-aminobutyric-acid type B receptor; CASPR2, contactin-associated protein-like 2; CSF, cerebrospinal fluid; NLR, neutrophil-to-lymphocyte ratio; EEG, electroencephalogram; MRI, magnetic resonance imaging.

Bold entries indicate p < 0.05.

**Table 3:** Univariate analysis of good and poor prognosis in anti-NMDAR encephalitis.

Variables	Total (n =48)	Good prognosis (n =42)	Poor prognosis (n =6)	*P*-value
Age (years), *M*(*IQR*)	34(19-44)	29.5(16.75-36.5)	19(15.5-41)	0.492
Sex, *n*(*%*)				0.402
Male	19(39.6)	16 (38.1)	3 (50)	0.911
Female	29(60.4)	26(61.9)	3 (50)	
Tumor, *n*(*%*)	4(8.3)	4 (9.5%)	0 (0)	1.000
Clinical manifestation				
Seizures, *n*(*%*)	34(70.8)	29 (69.0)	5 (83.3)	0.810
Cognitive deficits, *n*(*%*)	12(25.0)	11 (26.2)	1 (16.7)	1.000
Psychiatric and behavior disorders, *n*(*%*)	31(64.6)	27 (64.3)	4 (66.7)	1.000
Disturbance of consciousness, *n*(*%*)	20(41.7)	16 (38.1)	4 (66.7)	0.376
Movement disorders, *n*(*%*)	11(22.3)	9 (21.4)	2 (33.3)	0.897
Autonomic dysfunction, *n*(*%*)	7(14.6)	6 (14.3)	1 (16.7)	1.000
Speech disorder, *n*(*%*)	7(14.6)	6 (14.3)	1 (16.7)	1.000
Pulmonary infection complications, *n*(*%*)	4(8.3)	2 (4.8)	2 (33.3)	0.071
Mechanical ventilation, *n*(*%*)	8(16.7)	5 (11.9)	3 (50.0)	0.079
Admission to neurology intensive care unit, *n*(*%*)	6(12.5)	4 (9.5)	2 (33.3)	**0.029**
CSF findings				
Increased intracranial pressure, *n*(*%*)	16(33.3)	14 (33.3)	2 (33.3)	1.000
Increased white blood cell count, *n*(*%*)	34(70.8)	31 (73.8)	3(50.0)	0.471
Increased protein in CSF, *n*(*%*)	12(25.0)	12 (28.6)	0 (0)	0.313
NLR, *M*(*IQR*)	2.56(1.93-3.98)	2.50(1.81-3.64)	7.38 (2.05-21.24)	**0.043**
Albumin, *M*(*IQR*)	39.65(35.45-43.78)	40.3(36.45-44.15)	30.3(16.85-42.35)	**0.034**
Abnormal EEG, *n*(*%*)	30(62.5)	26 (61.9)	4 (66.7)	1.000
Abnormal MRI, *n*(*%*)	25(52.1)	23 (54.8)	2 (33.3)	0.585
Time to immune therapy (d), *M*(*IQR*)	21(11.25-54.25)	18.5 (11-48.75)	26 (17.25-413.75)	0.417

M,median; IQR, Interquartile Range; NMDAR, N-methyl-D-aspartate receptor; CSF, cerebrospinal fluid; NLR, neutrophil-to-lymphocyte ratio; EEG, electroencephalogram; MRI, magnetic resonance imaging.

Bold entries indicate p < 0.05.

**Table 4:** Multivariate analysis of factors associated with a poor prognosis.

	*β*	*SE*	*Wald*	*OR*	95%*CI*	*P*
NLR	0.318	0.116	7.558	1.374	1.096, 1.724	0.006
Tumor	2.434	0.758	10.318	11.408	2.583, 50.383	0.001
constant	-3.480	0.653	28.420	0.031	–	<0.001

SE, standarderror; OR, odds ratios; CI, confidence intervals; NLR, neutrophil-to-lymphocyte ratio.

**Figure 5:**
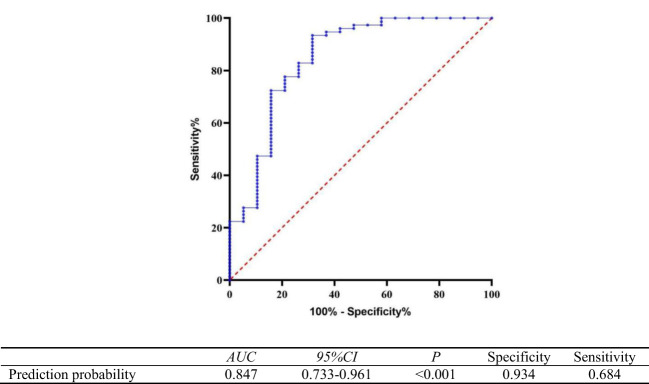
Receiver operating curve of the predictive value of the regression model equation for poor prognosis of autoimmune encephalitis.

The prognosis was considered the state variable (poor prognosis=1, good prognosis=0), and the prediction probability generated by the regression model equation was used as the independent variable for ROC analysis. The results showed that the area under the curve (AUC) of the prediction probability was 0.847 (95% CI,0.733-0.961; *p* < 0.001), with a specificity of 0.934 and a sensitivity of 0.684. The Hosmer–Lemeshow test showed that the regression model had a good calibration degree (χ2 = 3.283, DF=8,P=0.915), as shown in **Figure 5**.

## 4 Discussion

In this retrospective study, we collected and analysed the clinical data of AE in a sample of 103 patients with multiple AE subtypes. Furthermore, we compared differences in clinical features, auxiliary examinations, treatments, and prognosis for each subtype and analysed the factors affecting the prognosis of AE.

AE can be divided into two categories according to the location of the target antigen: classical paraneoplastic syndromes with antibodies targeting intracellular proteins (e.g. anti-Hu, anti-Yo, and anti-Ri) and encephalitis with antibodies against cell-surface neuronal receptors or synaptic proteins. The four antibodies included in our study belong to the latter category (7). Among all registered patients with AE, anti-NMDAR encephalitis accounted for the largest proportion. According to previous studies, anti-NMDAR encephalitis often occurs in young women. Our results were consistent with these findings. The onset ages of anti-GABA_B_R, anti-LGI1 and anti-CASPR2 encephalitis were relatively older compared to anti-NMDAR encephalitis, and men accounted for more cases. This result is consistent with the previous statistics. Notably, 96.4% (27/28) of patients with anti-LGI1 encephalitis in our cohort were males, which is higher than the 60–70% reported in other studies (8, 9). This may be attributed to our small sample sizes.

In our study, the most common symptoms of anti-NMDAR encephalitis were seizures (70.8%), psychiatric and behavior disorders (64.6%) and disturbance of consciousness (41.7%), which was consistent with other studies (10–12). Previous studies have shown that FBDS and hyponatraemia are the characteristic symptoms of anti-LGI1 encephalitis. The proportions of patients with FBDS and hyponatraemia in our cohort were 21.4% and 10.7%, respectively, which were lower than the 30–70% and 25–80% reported in other studies (8, 13, 14). This may be related to our small sample size. FBDS is a unique symptom of anti-LGI encephalitis. Therefore, the diagnosis should be highly suspected in any patient with FBDS, and antibody testing should be actively carried out (13). Previous studies have shown that patients with anti-CASPR2 encephalitis demonstrate a wide range of symptoms, including central nervous system symptoms, such as encephalopathy, and peripheral symptoms, such as peripheral nerve hyperexcitability/neuromyotonia (myokymia, fasciculations, cramps) and neuropathic pain (15, 16). In our study, only one patient with anti-CASPR2 encephalitis showed peripheral nerve hyperexcitability, which manifested as involuntary shaking of the head and limbs. This may be attributed to the small sample size. In our cohort, the most common clinical symptom of anti-GABA_B_R encephalitis was seizures (90%), all of which were manifested as generalized tonic-clonic seizures (GTCS). This is comparable to the 90–100% reported in other studies (17, 18).

Tumours are a common complication of AE. In our study, 4 patients with anti-NMDAR encephalitis had tumours, all of which were ovarian teratomas. They were all women of reproductive age, ranging from 15–35 years. Wu et al. summarised all studies on ovarian teratoma-related anti-NMDAR encephalitis from 2007 to 2020 (19). The incidence of anti-NMDAR encephalitis complicated by a teratoma was 37.4% in women, which is higher than the 13.8% reported in our cohort. Other single-centre studies in Asia have reported a relatively low teratoma prevalence in women with anti-NMDAR encephalitis, such as 13.3% reported by Lim et al. (11) and 14.3% reported by Huang et al. (12), which is consistent with our results. This may be related to the relatively small sample size and genetic backgrounds of the different races. However, the pathogenesis of anti-NMDAR encephalitis induced by ovarian teratomas remains unclear. Chefdeville et al. suggested that the glial tissue in teratomas might trigger or sustain the anti-tumour response associated with autoimmune neurological diseases (20). Anti-NMDAR encephalitis with other types of tumours is rare. Neuroblastoma, Hodgkin’s lymphoma, breast carcinoma, thymic carcinoma, pancreatic cancer, and lung cancer have also been reported in other studies (10, 21). Anti-GABA_B_R encephalitis often has a high tumour incidence, and the most common tumour type is SCLC (18). The incidences of tumour in anti-LGI and anti-CASPR2 encephalitis were relatively low. A small number of patients have been reported to have thymoma or lung cancer; however, this was not observed in our study (22).

The proportion of abnormal inflammation in the CSF differed among different antibody types. Studies by Marc Durr et al. showed that 94% of patients with anti-NMDAR encephalitis had abnormal CSF, whereas only 36% of patients with anti-LGI1 encephalitis had abnormal CSF (23). Blinder and Jan Lewerenz et al. reported that patients with antibodies against NMDAR, GABA_B_R, and AMPAR showed rather frequent inflammatory CSF changes, whereas the CSF of patients with antibodies against CASPR2, LGI1, GABA_A_R, or glycine receptors were found to be mostly normal (24). In our cohort, 52.2% of patients had CSF pleocytosis (70.8% for anti-NMDAR encephalitis,18.5% for anti-LGI1 encephalitis, 66.7% for anti-GABA_B_R encephalitis and 16.7% for anti-CASPR2 encephalitis). Anti-NMDAR and anti-GABA_B_R encephalitis had a higher proportion of CSF abnormalities compared with anti-LGI1 and anti-CASPR2 encephalitis, which is consistent with the results of previous studies.

In this study, 45.6% of patients with AE had abnormal MRI signals. Different antibody types showed different imaging manifestations. Previous studies have shown that 40–70% of patients with anti-NMDAR encephalitis have normal MRI results (Mueller et al.43%, Dalmau,50%, Titulaer, 66%, 10, 25, 26). The lesions and abnormal MRI findings in patients with anti-NMDAR encephalitis are often nonspecific, and the involved sites are scattered and variable, including the temporal lobe, hippocampus, basal ganglion, thalamus, cortex, and brainstem. Anti-LGI1, anti-CASPR2, and anti-GABA_B_R encephalitis mainly showed a T2-FLAIR hyperintense signal in the medial temporal lobe or the hippocampus (8, 13, 14). Our findings are consistent with these studies. In patients with normal MRI findings or lack of specific imaging findings, brain ^18^F-fluoro-deoxy-glucose positron emission tomography (FDG PET) can be considered (27, 28). An increasing number of studies have confirmed the high sensitivity of ^18^F-FDG PET for the diagnosis of AE. Anti-NMDAR encephalitis often presents as hypermetabolism in the basal ganglia and frontal lobe and significant hypometabolism in the occipital lobe (29). Hypermetabolism in the basal ganglia and medial temporal lobe is common in anti-LGI1 and anti-CASPR2 encephalitis (30, 31). This test was not conducted in our study due to equipment and financial problems; however, it can be considered for the early diagnosis of AE in future studies.

Currently, AE treatment includes first-and second-line immunotherapy, alternative therapy, and long-term maintenance therapy (32). In our cohort, 102 patients received first-line immunotherapy including corticosteroids and/or IVIG. None of the patients in our study received plasma exchange owing to the limitations of the medical devices. The first-line efficacy rates of anti-LGI1 encephalitis and anti-CASPR2 encephalitis were 92.3% and 83.3%, respectively, which is consistent with the findings of Teng et al. and Guo et al. (13, 14). Previous studies have reported a 60–80% response rate of anti-GABA_B_R encephalitis to immunotherapy, consistent with our results (33–35). The first-line efficacy rate for anti-NMDAR encephalitis was 70.2%, which was higher than that reported by Titulaer et al. (251/472,53%) (10). This may be related to the higher proportion of first-line combined immunotherapies using corticosteroids and IVIG (77% vs. 44%, respectively). Patients with poor response to first-line immunotherapy should start second-line treatment, including rituximab and/or cyclophosphamide. In our study, rituximab was administered to 21 patients as second-line immunotherapy. Similar to the response to first-line treatment, the efficacy rates of RTX in patients with anti-NMDAR and anti-GABA_B_R encephalitis were relatively low at 64.3% and 50%, respectively, whereas the efficacy rates of the other two antibody types were 100%. Rituximab is an effective drug for the treatment of severe and refractory AE, which is consistent with previous studies (10, 36). Additionally, some retrospective studies have reported the positive effects of tocilizumab and bortezomib (32, 37, 38). In our study, 5 patients with poor effect of second-line treatment received bortezomib. They all had a good prognosis (mRS ≤ 2) at the last follow-up. The abovementioned treatment have been widely accepted and implemented; however, no large-sample clinical randomised-controlled trials have been conducted to verify these methods.

The proportions of relapse for anti-NMDAR, anti-LGI1, anti-GABA_B_R, and anti-CASPR2 encephalitis were 14.6%, 4%, 63.2%, and 14.3%, respectively, at the last follow-up. The AE recurrence rate may be related to antibody subtypes, sample size, genetic background, follow-up time, and immunotherapy regimen (39). According to the results of the last follow-up, 78(78.8%) patients had a good prognosis (mRS 0–2), and 21(21.2%) had a poor prognosis (mRS 3–6). Although the neurological functional outcomes of most patients were good, the prognosis of the different subtypes differed. In our study, the proportions of patients with a good prognosis for anti-NMDAR, anti-LGI1, anti-GABA_B_R, and anti-CASPR2 encephalitis were 87.5%, 92.0%, 42.1%, and 71.4%, respectively. The proportion of patients with poor prognosis was significantly higher in anti-GABA_B_R encephalitis compared to other AE encephalitis subtypes (*p*<0.001). These results are consistent with previous studies (10, 11, 14, 15, 17, 18).

We further analysed the factors affecting the prognosis of patients with AE. Univariate analysis indicated that tumour presence (*p<*0.001), anti-GABA_B_R encephalitis (*p<*0.001), disturbance of consciousness (*p=*0.045), pulmonary infection complications (*p=*0.039), mechanical ventilation (*p=*0.030), elevated NLR (*p<*0.001), and low albumin levels (*p=*0.003) were statistically significant. In addition, subgroup analysis showed that elevated NLR, low albumin level, and ICU admission were risk factors for poor prognosis of anti-NMDAR encephalitis; however, we did not conduct a multivariate analysis of prognosis in each subgroup due to the limited sample size. Multivariate analysis of patients with AE suggested that elevated NLR and tumour presence were independent predictors of prognosis. NLR is a clinically relevant biomarker of pathological inflammation and can be obtained from the whole blood cell count, which is cheap and easily available. NLR reflects the relationship between the innate immune system (mediated by neutrophil granulocytes) and the adaptive immune system (mediated by lymphocytes). A high NLR is associated with a severe inflammatory response and reflects an imbalance between the innate and adaptive immune systems (40). Previous studies have confirmed that NLR is related to the prognosis of patients with cancer (41). In recent years, the NLR has been shown to be associated with the outcomes of patients with an increasing number of diseases, such as atherosclerosis, neurodegenerative diseases, and autoimmune diseases. Christopher et al. showed that a higher NLR strongly predicts increased multiple sclerosis (MS)-related disabilities (42). In our study, patients with a higher NLR at the onset of the disease were more likely to have poor neurological function at the last follow-up (OR, 1.374; 95% CI [1.096–1.724]; *p*=0.006). The risk of poor prognosis increased by 1.374 times for every 1 unit increase in the NLR, which is consistent with a previous study by our team that showed that elevated NLR could affect the response to first-line treatments (43). In addition to NLR, tumour presence was another independent predictor of poor prognosis (OR, 11.408; 95% CI [2.583-50.383]; *p*=0.001). In our study, six of the twelve patients (50%) who underwent anti-tumour therapy had a good prognosis and six died (50%), while all three patients without anti-tumour treatment died (100%). Although tumour presence predicts a poor prognosis, aggressive anti-tumour therapy can effectively improve the outcome. The regression equation of the model was logit(P)=-3.480 + 0.318 NLR+2.434 with or without tumours (with assignment=1, without assignment=0). The information provided by this equation can aid clinicians in identifying patients with poor prognosis in the early stages and providing more aggressive treatment options and a closer follow-up.

Our study had several limitations. First, this was a retrospective study; therefore, some confounding factors may have been challenging to eliminate, and certain examination data may have not been accessible. Second, the limited sample size in our single-centre study may have introduced a potential bias in the results. Furthermore, the restricted sample size prevented us from conducting separate prognostic analyses for each AE subtype. Third, none of the patients in our study received plasma exchange owing to the limitations of the medical devices, and only a few patients received RTX. This may have led to bias in our statistical results, which should be verified in larger randomised controlled studies. Finally, the mRS score has limitations in the evaluation of neurological function in AE; therefore, it is necessary to develop a more detailed and reasonable scoring scale.

In conclusion, our study described, compared, and analysed the clinical features, treatment, and prognosis of patients with anti-NMDAR, anti-GABA_B_R, anti-LGI1 and anit-CASPR2 encephalitis. Different antibody subtypes demonstrated different clinical symptom spectra throughout the disease stages. Anti-LGI1 encephalitis and anti-CASPR2 encephalitis were more sensitive to first-line and second-line treatment. Anti-GABA_B_R encephalitis had the worst prognosis among the abovementioned subtypes. The regression equation constructed using NLR and tumour presence effectively predicted the poor prognosis of AE.

## Data availability statement

The datasets presented in this study can be found in online repositories. The names of the repository/repositories and accession number(s) can be found in the article/Supplementary Material.

## Ethics statement

The studies involving humans were approved by the Research Ethics Committee of Shandong Provincial Hospital. The studies were conducted in accordance with the local legislation and institutional requirements. Written informed consent for participation in this study was provided by the participants’ legal guardians/next of kin. Written informed consent was obtained from the individual(s), and minor(s)’ legal guardian/next of kin, for the publication of any potentially identifiable images or data included in this article.

## Author contributions

TH is responsible for experimental design. While FL, BW, CW and MH are responsible for the implementation of the experiment and the pictures of the article. SG is responsible for writing the article. All authors contributed to the article and approved the submitted version.
